# Universal Fiducial Markers for Multi-Modal Radiotherapy

**DOI:** 10.1016/j.ijpt.2026.101308

**Published:** 2026-02-10

**Authors:** Jie Liu, Caroline Bamberger, Naveed Iqbal, Kevin Reynolds, Smit Shah, Xing Li, Peng Wang, Jiajin Fan, Ashish Chawla, Kevin Choe, Daniel Kim, Robabeh Rahimi

**Affiliations:** 1 Inova Schar Cancer Institute, Inova Health System, Fairfax, VA, USA; 2 Medical Device Manufacturer, IZI Medical Products, Owing Mills, MD, USA; 3 University of Maryland School of Medicine, Baltimore, MD, USA

**Keywords:** Fiducial markers, Image-guided radiotherapy, Proton therapy, Proton beam perturbation, Image tracking

## Abstract

**Purpose:**

Integrating multiple radiotherapy modalities, including photon and proton therapy, within a single clinical facility requires universal fiducial markers that perform reliably across all imaging and treatment systems. For computed tomography (CT) and image-guided radiotherapy (IGRT), fiducials should be large enough to ensure visibility without introducing significant image artifacts. In CyberKnife treatments, a fiducial diameter of at least 0.75 mm is necessary to maintain accurate tracking, particularly in the abdominal and pelvic regions. However, fiducials of this size are unsuitable for proton therapy due to the considerable beam perturbation they cause. This study investigates the design and implementation of customized fiducials that balance imaging visibility, tracking reliability, and proton beam perturbation, enabling their use across multiple radiotherapy modalities.

**Materials/Methods:**

Customized coil-shaped platinum fiducials with a 0.75 mm diameter and variable coil gaps were developed to reduce the amount of metal in the proton beam path. For image visibility and trackability assessments, both standard fiducials (0.5 and 0.75 mm diameters with nominally zero coil gaps) and customized versions were inserted in an anthropomorphic phantom. Visibility was evaluated using cone-beam CT (CBCT) and kilovoltage (kV) imaging on both photon and proton treatment systems. Trackability was assessed in the CyberKnife system using an anthropomorphic phantom and a respiratory motion platform. Proton beam perturbation was measured using radiochromic films that were placed downstream of the fiducials and irradiated with a broad spread-out Bragg peak (SOBP) field. A triple-channel film dosimetry method was employed for analysis.

**Results:**

Customized fiducials with increased coil gaps demonstrated visibility comparable to standard fiducials in IGRT for both photon and proton systems. In the CyberKnife system, these customized fiducials also provided reliable tracking performance under both static conditions and simulated respiratory motion. The film measurements revealed that the customized fiducials with coil gaps larger than 0.5 mm/coil could significantly reduce proton beam perturbation compared to both the standard 0.75 and 0.5 mm diameter fiducials.

**Conclusions:**

This study demonstrates the feasibility of redesigning fiducial markers to maintain a large diameter for reliable imaging visibility and trackability while minimizing proton beam perturbation by increasing the coil gap. Adopting a universal fiducial marker design has the potential to streamline clinical workflows and support seamless integration across various radiotherapy modalities.

## Introduction

Image guidance in radiotherapy is essential for accurate patient positioning and precise tumor targeting. Fiducial markers are widely used in image-guided radiotherapy (IGRT) as reference landmarks to localize tumors in various treatment sites.^1^, ^2^, ^3^ Selecting appropriate fiducial markers is critical to achieving optimal IGRT, requiring a balance between sufficient visibility and minimal artifact production across different imaging systems.^4^, ^5^

Proton therapy is valued for its precision, providing conformal dose distribution with minimal exit dose.^6^, ^7^ However, implanted fiducial markers can cause dose perturbation in proton beams, leading to dosimetric uncertainties that may compromise treatment effectiveness.^8^, ^9^, ^10^, ^11^, ^12^, ^13^ While various strategies have been proposed to minimize these effects, they often add complexity to treatment planning. For example, a prior study on prostate treatment using multiple large gold fiducial markers demonstrated that more than two proton fields were needed to counteract the significant underdosing caused by the markers, without reducing tumor control probability.^12^

In contrast, photon therapy is generally more tolerant of dense metal fiducial markers through the use of multiple beam angles in advanced delivery techniques such as intensity modulated radiation therapy/volumetric modulated arc therapy, where larger markers are often used to ensure reliable imaging visibility or tracking. However, in centers that offer both proton and photon therapies, the use of various types of fiducials presents practical challenges—particularly when the final treatment modality has not been determined at the time of fiducial implantation. This uncertainty may be due to pending insurance approval or changes in the patient's suitability for proton therapy. In such cases, the use of a universal fiducial marker that is compatible with both treatment modalities is especially beneficial, as it allows for flexibility if a change in treatment approach becomes necessary.^14^

Recent innovations in fiducial marker design, such as liquid markers and polymer-encapsulated markers, seek to replace metal with low-density materials to minimize the perturbation effect in proton therapy.^15^, ^16^, ^17^, ^18^ Despite these advances, there remains a need for conventional metal fiducial markers that can perform consistently across all imaging and treatment modalities.^19^, ^20^, ^21^ This study aims to address this need by investigating customized coil-shaped platinum fiducial markers with increased coil gaps, designed to reduce the amount of metal material intersecting the proton beam path while maintaining adequate imaging visibility and trackability in IGRT.

## Materials and methods

### Fiducial fabrication

A range of fiducial marker products was evaluated based on criteria including design flexibility, imaging visibility, artifact production, proton beam perturbation, cost-effectiveness, and availability. The Visicoil fiducial marker (IZI Medical Products, Owing Mills, MD) was selected due to its coil-like design that is customizable by adjusting the coil gap or pitch value. Visicoil fiducial markers are made from biocompatible gold or platinum and are available with different sizes (diameter: 0.35, 0.5, 0.75, and 1.0 mm; length: 5 and 10 mm for platinum marker, and 5, 10, 20, and 30 mm for gold marker). The standard Visicoil marker features a nominally zero coil gap.

In our clinic, a large Visicoil platinum fiducial (0.75 mm diameter, 5 mm length) is routinely used for patients undergoing photon therapy, including those receiving fiducial-based tracking treatment with the CyberKnife system. Conversely, a small Visicoil platinum fiducial (0.5 mm diameter, 5 mm length) is used for patients receiving proton therapy in order to minimize beam perturbation caused by the marker.

To support multi-modality treatment, we collaborated with the manufacturer to develop customized versions of the large Visicoil fiducial by increasing the coil gap (0.25, 0.5, 0.75, 1.0, 1.25, and 1.5 mm per coil). These modifications reduced the amount of metal in the beam path, thereby decreasing the proton beam perturbation. At the same time, by maintaining the large 0.75 mm diameter, the customized markers were expected to preserve adequate visibility and trackability for photon-based imaging and treatment.

Table 1 summarizes the specifications of the 2 standard and 6 customized fiducial markers evaluated in this study.

**Table 1:** The physical specifications of the Visicoil platinum fiducial markers used in this study

	Type	Length (mm)	Diameter (mm)	Gap (mm)	Mass (gram/coil)
Fiducial 1	Customized	5	0.75	1.5	0.008
Fiducial 2	Customized	5	0.75	1.0	0.0093
Fiducial 3	Customized	5	0.75	0.25	0.0266
Fiducial 4	Customized	5	0.75	1.25	0.009
Fiducial 5	Customized	5	0.75	0.5	0.016
Fiducial 6	Customized	5	0.75	0.75	0.012
Fiducial 7	Standard	5	0.75	0*	NA*
Fiducial 8	Standard	5	0.50	0*	NA*

The fiducials are numbered to be consistent with their order in the radiochromic film measurements for proton beam perturbation evaluation.

* : the standard coil gap is nominally zero, the mass not provided by the manufacturer.

### Phantom preparation

**Figure 1:**
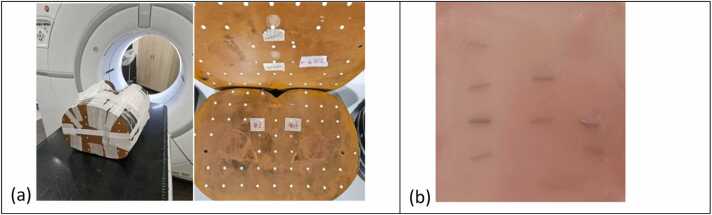
(a) Rando phantom for fiducial visibility and trackability assessments, with the fiducials inserted into the small holes in multiple phantom slabs (only 2 fiducial inserts shown on the picture). (b) Wax sheet with fiducials embedded and laid flat, used for the proton beam perturbation measurement.
(2) Wax Sheet Phantom for Proton Beam Perturbation study: A thin wax sheet (thickness 1.5 mm) was fabricated to align and secure the 8 fiducial markers on a plane (Figure 1b). The fiducial markers were gently placed on the melted wax surface, and the top side was flipped down to orient the markers toward the first film. This wax sheet was supported by a plastic frame when placed under solid water phantoms for the perturbation measurement.

Two phantoms were used in this study:

(1) Rando Phantom for Clinical Imaging Assessments: To evaluate the imaging visibility and trackability of fiducial markers under clinical conditions, an anthropomorphic Rando phantom was utilized (Figure 1a). Each of the 8 fiducial markers was inserted into a small hole within the phantom slabs and secured using wax. The markers were distributed across 4 slabs located in the pelvic region, with 2 markers per slab. To prevent overlapping on kV orthogonal image pairs, the markers were positioned with adequate spacing between them.

### Imaging visibility and trackability assessment

The imaging visibility of the fiducial markers was evaluated using cone-beam computed tomography (CBCT) and kV planar images acquired on a linear accelerator (TrueBeam, Varian Medical Systems, CA) and a proton treatment system (ProteusPlus, Ion Beam Applications [IBA] Proton Therapy, Belgium). Imaging parameters, including kV, mA, and exposure time (ms), were adjusted according to the clinical protocols specific to each system. Two independent observers assessed the visibility of each fiducial marker on the acquired images. For each image, the visibility of the marker was rated as either clinically acceptable or not acceptable based on clarity and ease of identification. In addition, the contrast-to-noise ratio (CNR) of each fiducial marker was quantitatively calculated on the kV and CBCT images by measuring the mean pixel intensity within the marker and a surrounding background region and dividing the intensity difference by the standard deviation of the background value.

Fiducial trackability was evaluated in the CyberKnife system (Accuray, WI) in fiducial tracking mode. To simulate tracking in anatomical sites with infrequent motion (e.g., prostate cancer), tests were performed with a static Rando phantom. To simulate tracking with respiratory motion, the Rando phantom was placed on a QUASAR respiratory motion platform (Modus Medical Devices Inc, Ontario, Canada) programmed with sinusoidal motion, and the CyberKnife Synchrony real-time tracking system was employed. Imaging parameters were selected within the standard clinical range for pelvic and abdominal treatments.

For the static Rando phantom, fiducial trackability was quantified using the tracking uncertainty values reported by the CyberKnife system for each marker. This uncertainty, calculated by the system’s fiducial extraction algorithm, represents the likelihood of an incorrect fiducial location. If the uncertainty exceeds a user-defined threshold, treatment delivery is interrupted with a soft stop. In our clinical practice, this threshold is typically set between 40% and 60%, depending on the reliability of fiducial visualization in kV images.

For tracking with respiratory motion, the Synchrony system acquires multiple live images to correlate fiducial positions with respiratory phases measured by external LED markers. The tracking was deemed successful if a stable and accurate correlation model would be maintained throughout the entire respiratory cycle.

### Proton beam perturbation evaluation

**Figure 2:**
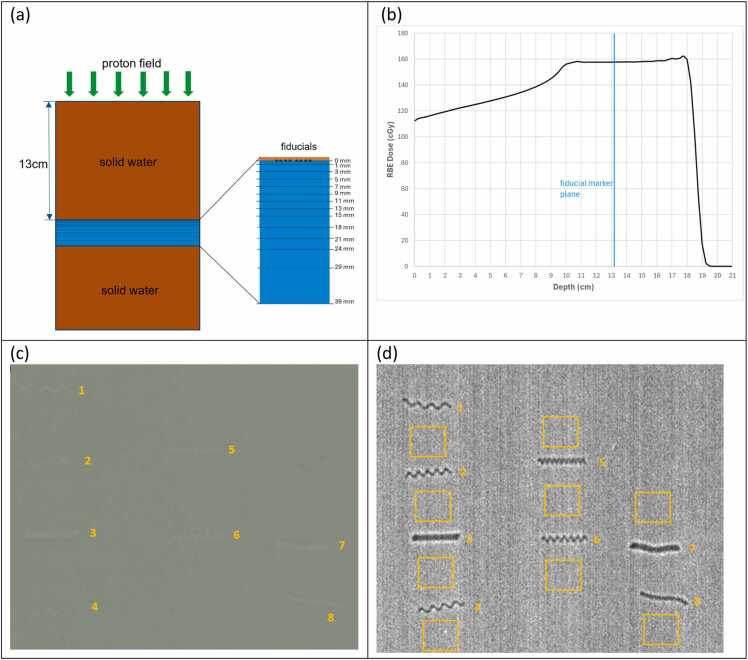
Proton Beam Perturbation Evaluation: (a) Schematic of measurement setup, with the central layer shown in an enlarged view, that contains the fiducials and radiochromic films embedded between solid water slabs. (b) Planned depth dose of the proton field, where each energy layer has an uniform spot spacing and the same MU per spot. (c) The irradiated film at depth 7 mm, with the fiducial projection positions labelled. (d) The corresponding dose map using the triple channel film dosimetry method.

Figure 2a illustrates the measurement setup using solid water phantoms, the fiducial marker wax sheet, and radiochromic films. Gafchromic EBT3 radiochromic film (Ashland Advanced Materials, Bridgewater, NJ) was used to measure the dose reduction caused by the presence of fiducial markers in the proton beam. Multiple films were placed at sequential depths ranging from 0 to 39 mm downstream of the markers, with solid water slabs used to separate each film. The incident proton field had a spread-out Bragg peak (SOBP) with 18 cm range and 8 cm modulation (R18M8), and 15 x 15 cm^2^ field size. The proton field comprised 27 energy layers with energy ranging from 114 to 164 MeV. The proton plan was delivered using the pencil-beam scanning technique on the IBA Proteus system. A 13 cm solid water phantom was placed upstream of the wax sheet. To ensure uniform dose distribution across the planes of fiducial markers and films, the proton field was planned with equal spot spacing and the same MU per spot for each energy layer, while the SOBP flatness was compromised to be around 5% (Figure 2b).

Radiochromic films from the same production batch were calibrated using data from all 3 color channels (red, green, and blue) for performing triple-channel film dosimetry analysis as described in reference.^22^ The calibration curves were fitted using a logarithmic rational function of the form:

$${\mathrm{OD}}_{k}=-\log(\frac{a_{k}+b_{k}∙\mathrm{dose}}{c_{k}+\mathrm{dose}})$$

where OD represents optical density, *k* indicates the color channel (red, green, blue), and ($a_{k},\;b_{k},\;c_{k})\;$are the fitting parameters. The resulting calibration curves are shown in Supplement S1.

Each irradiated film (Figure 2c) was converted into a dose map (Figure 2d). For each fiducial projection, the mask corresponding to the fiducial’s shape was extracted, and the average dose across all pixels within that mask was calculated. Additionally, nine reference regions, defined as areas free from fiducial influence, were selected around each projection, and their average dose was defined as the reference dose in each depth.

The proton beam perturbation caused by each fiducial at each depth was then quantified as the relative dose reduction in the fiducial projection compared to the reference dose.

The measurement depths were converted to water-equivalent thicknesses. The solid water phantom (SP34, IBA Dosimetry GmbH, Schwarzenbruck, Germany) used in this study has a water-equivalent ratio of 1.04. The EBT3 radiochromic film has a physical thickness of 0.28 mm and a water-equivalent thicknesses of 0.37 mm (Ref.^23^), with its active layer located at the central plane. Accordingly, the water-equivalent depths for the dose measurements were 0.185, 1.595, 4.045, 6.495, 8.945, 11.395, 13.845, 16.295, 18.745, 22.235, 25.725, 29.215, 34.785, and 45.555 mm.

## Results

### Imaging visibility and trackability

**Figure 3:**
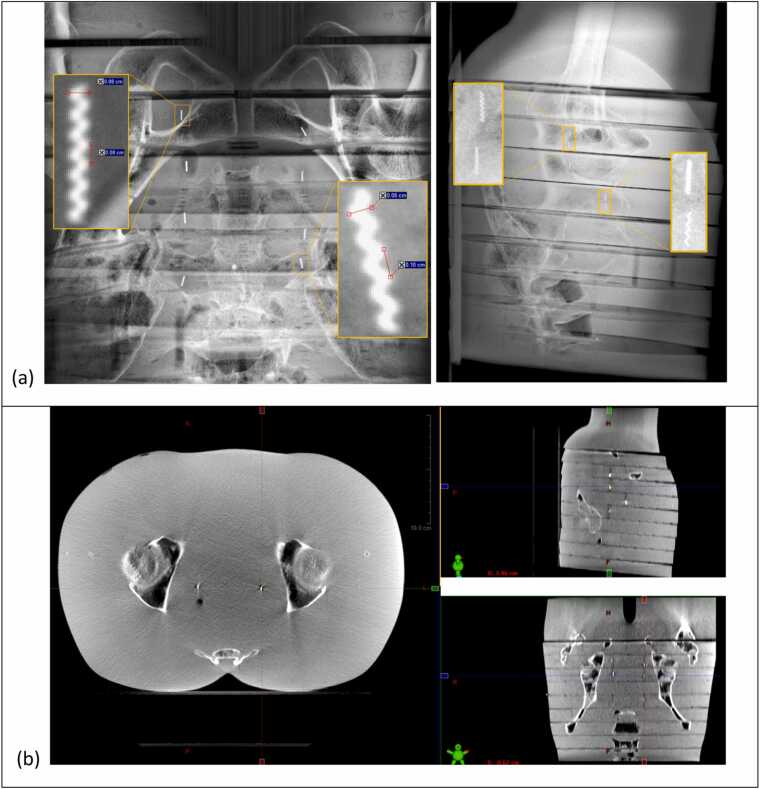
Imaging visibility assessment using the Rando phantom: (a) kV portal and orthogonal images acquired on the IBA ProteusPlus proton system (Imaging parameters: 120 kV/320 mA/320 ms). Enlarged views are displayed for selected fiducial markers (kV portal: #6 (left), #2 (right); kV orthogonal: #6, #8 (left), #5, #4 (right), with fiducial numbering from Table 1.). (b) CBCT image acquired on the IBA ProteusPlus proton system (CBCT protocol parameters: 125 kV/320 mA/4.83 s) All fiducial markers can be readily identified.

All customized fiducial markers demonstrated acceptable visibility in IGRT, including CBCT and kV imaging, for both photon and proton delivery systems. Figure 3a and b show kV pair images and CBCT, respectively, acquired on the IBA proton system using the Rando phantom. The pelvis imaging protocol was applied for both imaging techniques. All 6 customized fiducials, along with the 2 standard fiducials, were clearly visible on both the kV pair and CBCT images. For the selected fiducials shown in the magnified view on Figure 3a, the measured CNRs were 33.4 for the left fiducial and 27.7 for the right fiducial on the kV portal image and 7.7-7.8 (left fiducials) and 9.6-9.8 (right fiducials) on the kV orthogonal image. The customized fiducial marker with the largest coil gap (1.5 mm) is highlighted on the CBCT image, with a measured CNR of 18.7. Comparable results (Supplement S2) were observed in the kV and CBCT images acquired on the Truebeam linear accelerator.

**Figure 4:**
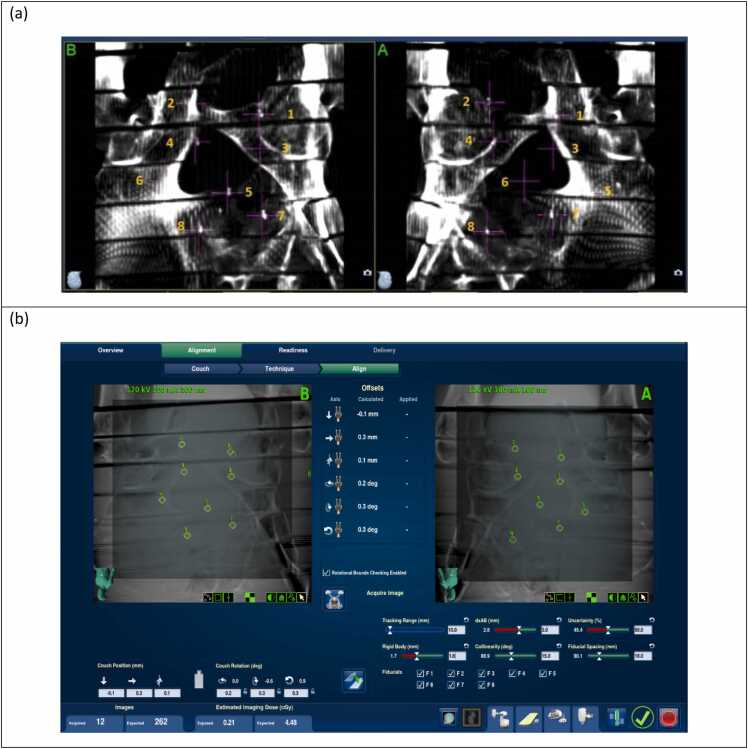
Imaging Visibility Assessment using the Rando phantom: (a) kV pair DRRs generated on the CyberKnife treatment planning system, with each fiducial marked by a magenta cross. (b) kV pair images acquired on the CyberKnife delivery system for alignment, with imaging parameters 120 kV/100 mA/100 ms. Yellow diamonds indicate the reference fiducial positions from the DRRs, and green circles indicate the detected fiducial positions on the live images. All 8 fiducials were successfully detected by the delivery system for alignment.

Figure 4(a) displays the kV pair Digitally Reconstructed Radiographs (DRRs) generated by the CyberKnife treatment planning system, demonstrating sufficient marker contrast on the CT images for the system to identify reference fiducial locations (marked by magenta crosses) for tracking. Figure 4(b) shows the kV pair images acquired on the CyberKnife delivery system, where the real-time auto detection of each fiducial (green diamonds) and their corresponding reference locations (yellow circle) are indicated.

**Figure 5:**
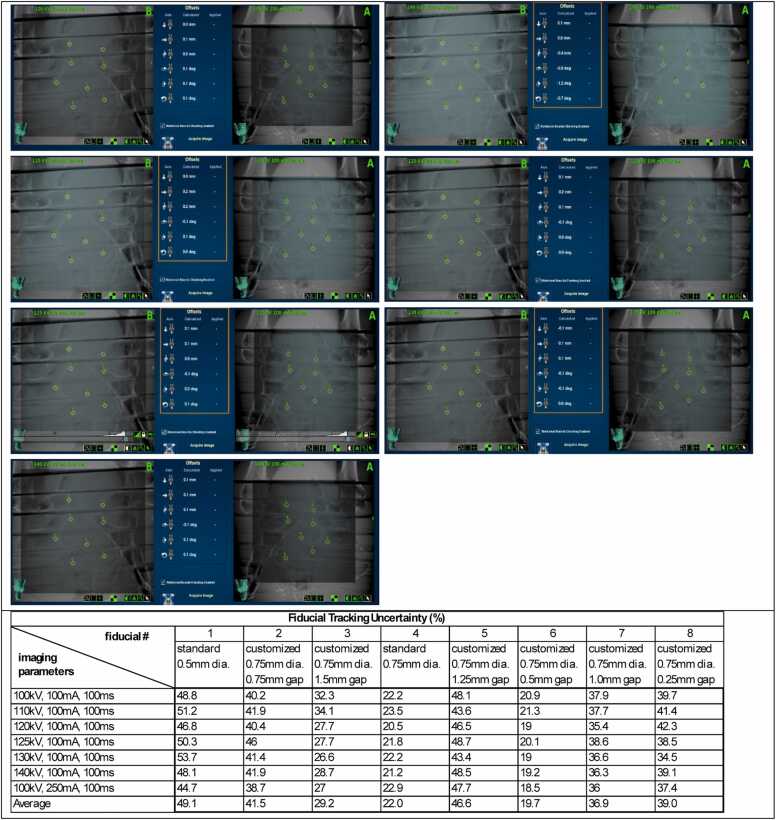
Fiducial tracking with the CyberKnife System: (top) Dynamic kV pair images showing successful tracking of each fiducial with different imaging parameters (bottom). Summary of the fiducial tracking uncertainties recorded from the CyberKnife treatment console. Note that the fiducial numbering shown here follows the order assigned by the CyberKnife planning system and is different from the numbering shown on Table 1, where the order corresponds to the proton perturbation measurements.

For imaging trackability testing on the CyberKnife system, each fiducial was reliably detected across various imaging parameters used in simulated tracking-based treatments (Figure 5, top). The tracking uncertainties for each fiducial, displayed on the treatment console during the simulated treatments, were recorded (Figure 5, bottom). Tracking uncertainty is a parameter calculated by the system during real-time treatment to indicate the quality of continuous fiducial tracking. Note that the fiducial numbering in the table shown in Figure 5 (bottom) corresponds to the CyberKnife system’s internal labeling and differs from the numbering presented in Table 1.

**Figure 6:**
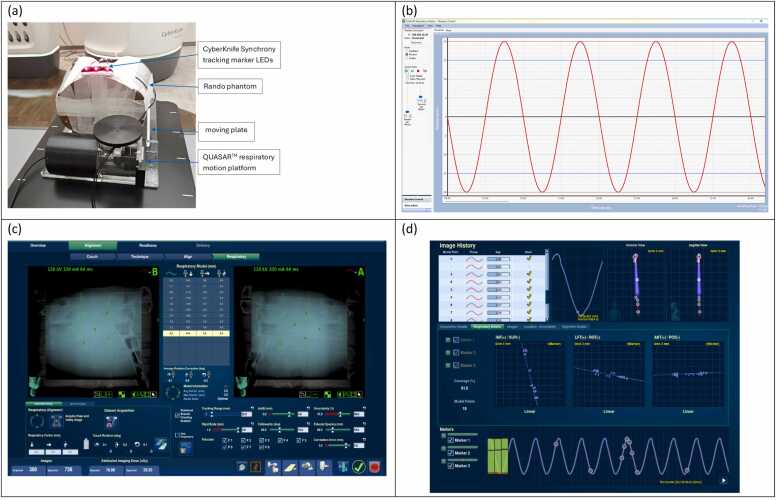
Fiducial tracking with respiratory motion in the CyberKnife system. (a) Measurement setup using the QUASAR respiratory motion platform and the Rando phantom. (b) Simulated sinusoidal motion with ±20 mm motion range. (c) Validated tracking of all 8 fiducials. (d) Correlation model between fiducials and external tracking markers, demonstrating stable trackability under the simulated respiratory motion.

Figure 6 presents the results of fiducial tracking with respiratory motion, as described in Method 3.3. With a simulated ±20 mm motion range along the superior-inferior direction, a stable correlation model between the eight fiducials and the Synchrony external LED markers was maintained throughout the entire respiratory cycle, confirming reliable trackability under the simulated motion.

### Proton beam perturbation

**Figure 7:**
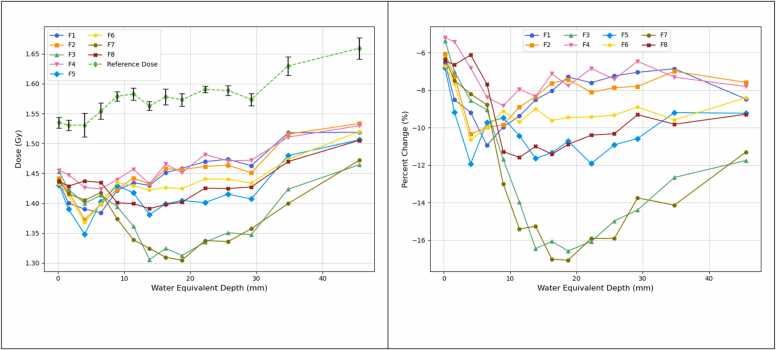
Proton beam perturbation: (a) The dose at each fiducial projection position (Fiducial 1-8, solid lines) as a function of water equivalent depth downstream of the fiducials. The reference dose at each depth is shown as the dashed line. Error bars represent the standard deviation of doses measured from nine reference ROIs on each film. (b) The percentage change in dose across each fiducial projection position relative to the reference dose of the same depth. Note the fiducial numbering order corresponds to that listed on Table 1.

Figure 7(a) presents the measured doses as a function of depth. Beam perturbation is indicated by decrease of dose at the fiducial projection position relative to the reference dose at the same depth, as shown in Figure 7b.

Across all fiducials, the relative dose exhibits a sharp decrease of approximately 9%-16% within the first 3-13 mm of depth, followed by a slight rebound before stabilizing at a consistent level at greater depths. In this region of consistent underdose, the standard 0.75 mm diameter fiducial (F7) and the customized fiducial with the smallest coil gap (0.25 mm, F3) show the greatest dose perturbation. The customized fiducial with 0.5 mm coil gap (F5) demonstrates dose perturbation comparable to the standard 0.5 mm diameter fiducial (F8). The other customized fiducials with larger coil gaps (0.75-1.5 mm; F1, F2, F4, and F6) exhibit reduced dose perturbation compared to the standard 0.5 mm diameter fiducial, with an improvement in underdose of approximately 2%.

## Discussion

The results from the proton beam perturbation measurements in this study confirmed the anticipated reduction in dose perturbation for the Visicoil fiducial marker achieved by increasing the coil gap while maintaining a large diameter of 0.75 mm. When the coil gap was increased to 0.5 mm or greater, the perturbation effect was found to be comparable or even lower than that of the standard fiducial with a smaller diameter (0.5 mm). To improve accuracy in measuring dose variation across the small fiducial projection area, a triple-channel film dosimetry method was employed. This approach, in contrast to single-channel analysis, helps reduce the influence of intrinsic non-uniformities in the film’s active layer.^22^

The coil-shaped Visicoil fiducial marker is small and irregular in shape, which results in relatively large fluctuations in pixel values across its projection areas on films. As a result, the variability observed on Figure 7 likely reflects the difficulty in accurately quantifying dose within the small projection areas. From our perturbation measurements, the variation of each fiducial curve is within approximately 1% in the plateau region.

Notably, as shown in Figure 7a, the reference dose exhibits noticeable variation with depth, which is consistent with the planned SOBP flatness used for irradiation. Because the dose perturbation was evaluated as a relative measurement where the dose in each fiducial projection area was normalized to the reference dose at the same depth, it was essential to maintain a uniform beam profile rather than a flat depth-dose across the SOBP. Supplement S3 shows the dose distribution of the proton plan, along with the depth dose curve across the central axis and beam profiles across the middle plane of SOBP. A high uniformity across the film measurement area (off-axis distance within 5 cm) was achieved.

The proton dose perturbation data presented in Figure 7 were based on single acquisitions, consistent with the feasibility nature of this study. Future work will involve repeated measurements to achieve statistical significance and a more comprehensive quantitative assessment of dose perturbation from the customized Visicoil markers. The imaging assessment of the customized Visicoil markers, conducted using the Rando phantom, demonstrated reliable visibility and trackability in clinical settings due to the preservation of a large marker diameter. However, the assessment was limited to qualitative evaluation by two independent observers and focused solely on the pelvic region of the Rando phantom. Further evaluation should include larger phantoms and different anatomical sites to improve generalizability.

Another limitation of the imaging trackability assessment was the reliance on a single quantitative parameter, the tracking uncertainty, provided by the CyberKnife delivery system. This metric is influenced not only by fiducial size and density but also by the contrast between the fiducial and surrounding tissue in the DRRs. As summarized in Figure 5, tracking uncertainty did not show a strong correlation with fiducial size, specifically in terms of coil gap. Future work will explore additional quantitative metrics for evaluating trackability to better inform the selection of optimal coil gaps.

This study did not aim to determine the optimal coil gap for the customized Visicoil markers. Instead, it focused on evaluating the feasibility of using customized markers that are compatible with both proton and photon therapies. From a practical standpoint, a moderate coil gap (0.5-1.0 mm) in the 0.75 mm diameter Visicoil marker appears to offer a balanced performance—minimizing proton beam perturbation while maintaining imaging visibility and trackability comparable to that of the standard large marker.

## Conclusion

This study demonstrated the feasibility of optimizing the Visicoil fiducial marker for use across multiple radiotherapy modalities. By increasing the coil gap, we were able to reduce proton beam perturbation while preserving the marker dimensions necessary for visibility and trackability in IGRT. These findings have important clinical implications, particularly in scenarios where multiple treatment modalities are available, and the final approach is not determined at the time of fiducial implantation. The ability to use a single, universal fiducial marker across different modalities could simplify treatment planning and enhance clinical efficiency. This is especially valuable in proton therapy, where minimizing beam perturbation is essential for maintaining dose integrity and ensuring effective treatment. Further clinical validation is needed to confirm these results across a broader range of treatment settings. Long-term studies assessing the stability and performance of customized fiducials in vivo will provide additional insight into their clinical utility.

## Declaration of Competing Interest

The authors declare the following financial interests/personal relationships, which may be considered as potential competing interests: Robabeh Rahimi reports equipment, drugs, or supplies were provided by IZI Medical Products. Kevin Reynolds reports a relationship with IZI Medical Products LLC that includes: employment. Smit Shah reports a relationship with IZI Medical Products LLC that includes: employment. If there are other authors, they declare that they have no known competing financial interests or personal relationships that could have appeared to influence the work reported in this paper.

## Declaration of Generative AI and AI-Assisted Technologies in the Writing Process

During the preparation of this work, the authors used ChatGPT-4o in order to improve language and readability only. After using this tool/service, the authors reviewed and edited the content as needed and take full responsibility for the content of the publication.
