# Supplementary material for: Universal Fiducial Markers for Multi-Modal Radiotherapy
Source: Int J Part Ther. 2026 Feb 10;19:101308. doi: 10.1016/j.ijpt.2026.101308 (PMC12927308; doi:10.1016/j.ijpt.2026.101308)
Supplement: Supplementary file 1 — Supplementary material [file mmc1.docx]

**Supplement S1.** EBT3 radiochromic film calibration for triple channel film dosimetry analysis.


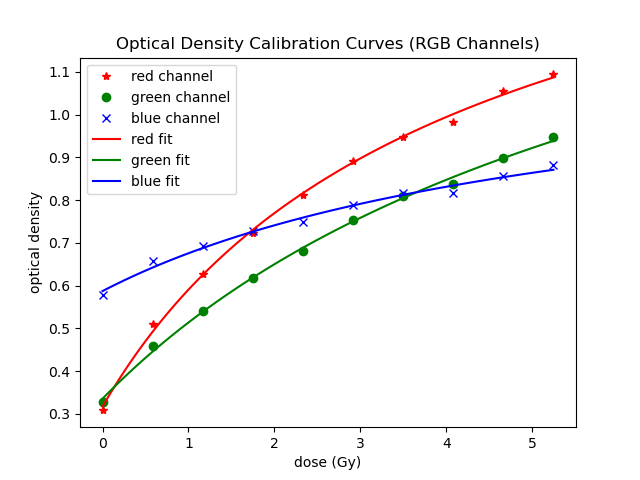


Figure S1. EBT3 radiochromic film calibration measurements and fitted curves using logarithmic rational function.

**Supplement S2.** Fiducial marker visibility tests on Truebeam® kV and CBCT.


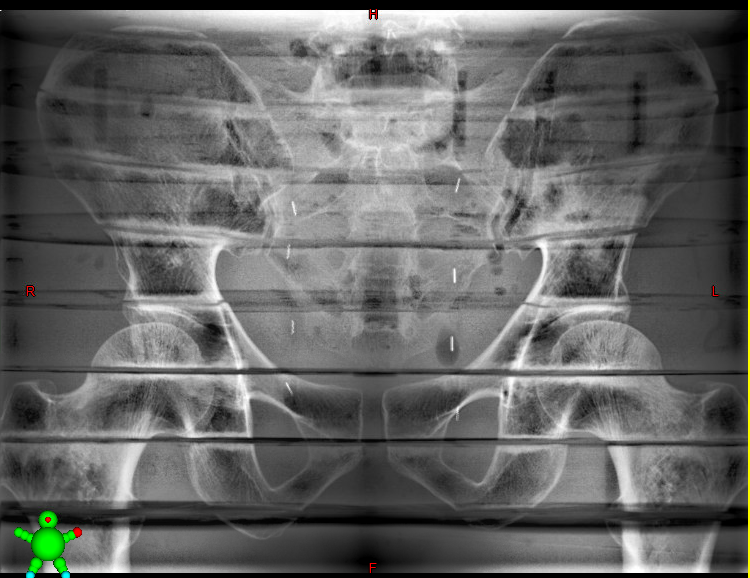

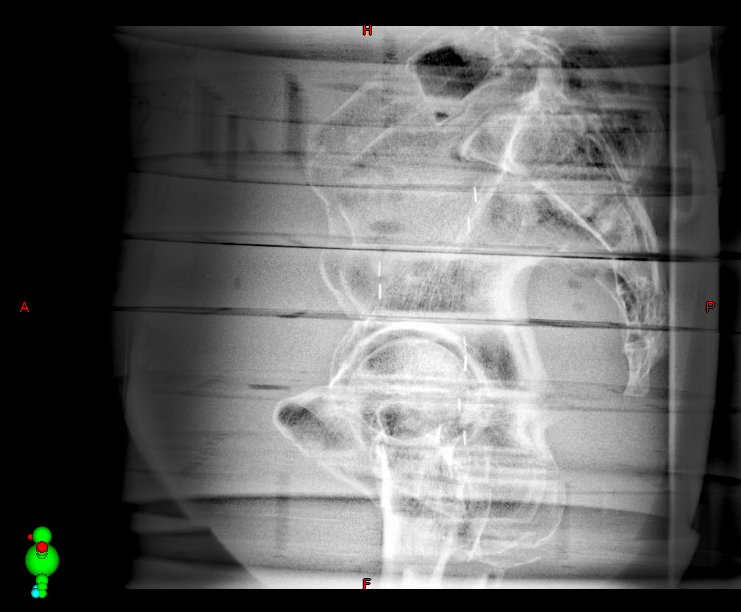


Figure S2.1. kV pair images acquired with Pelvis protocol on Truebeam® (imaging parameters: 100kVp/200mA/50ms).


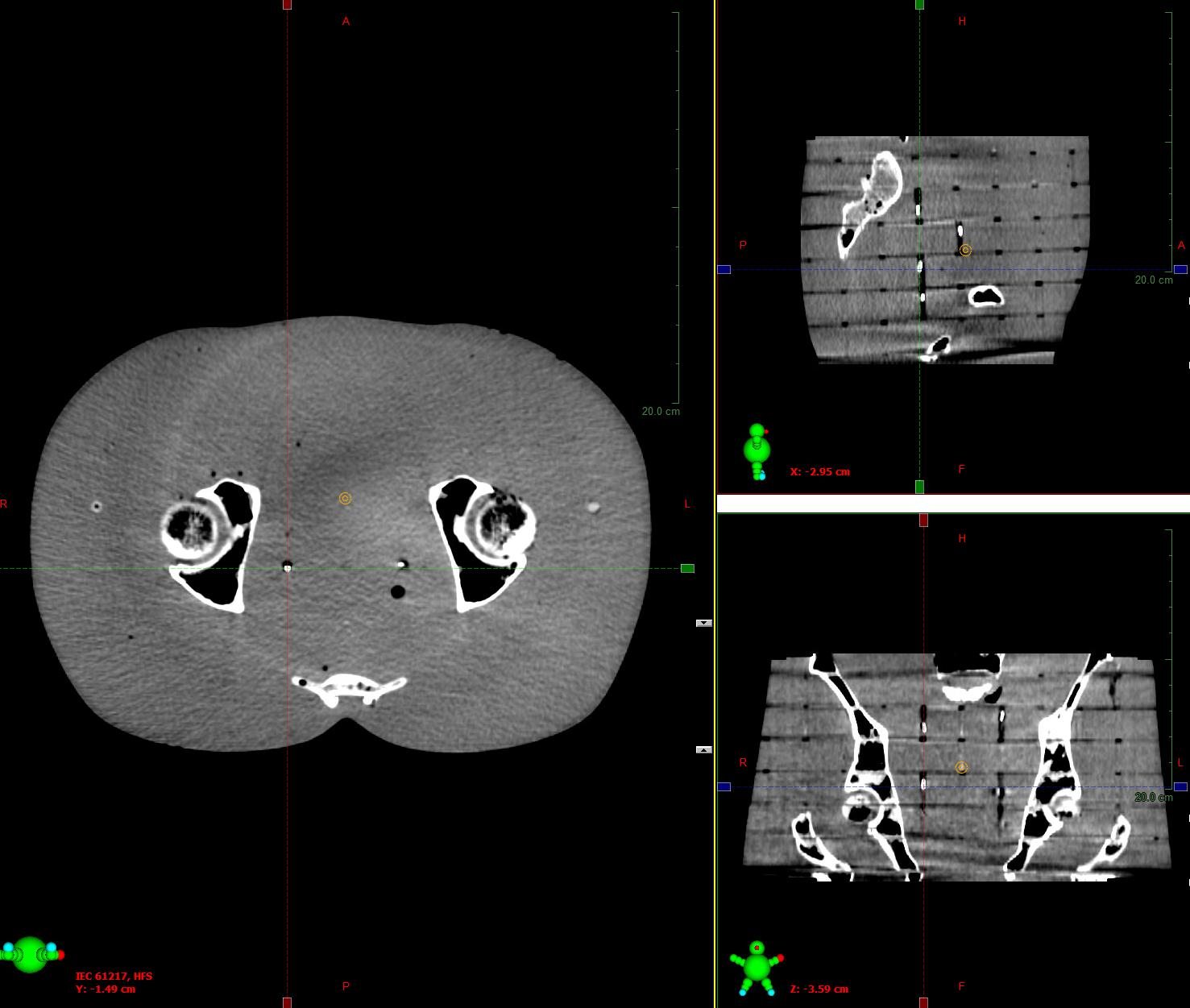

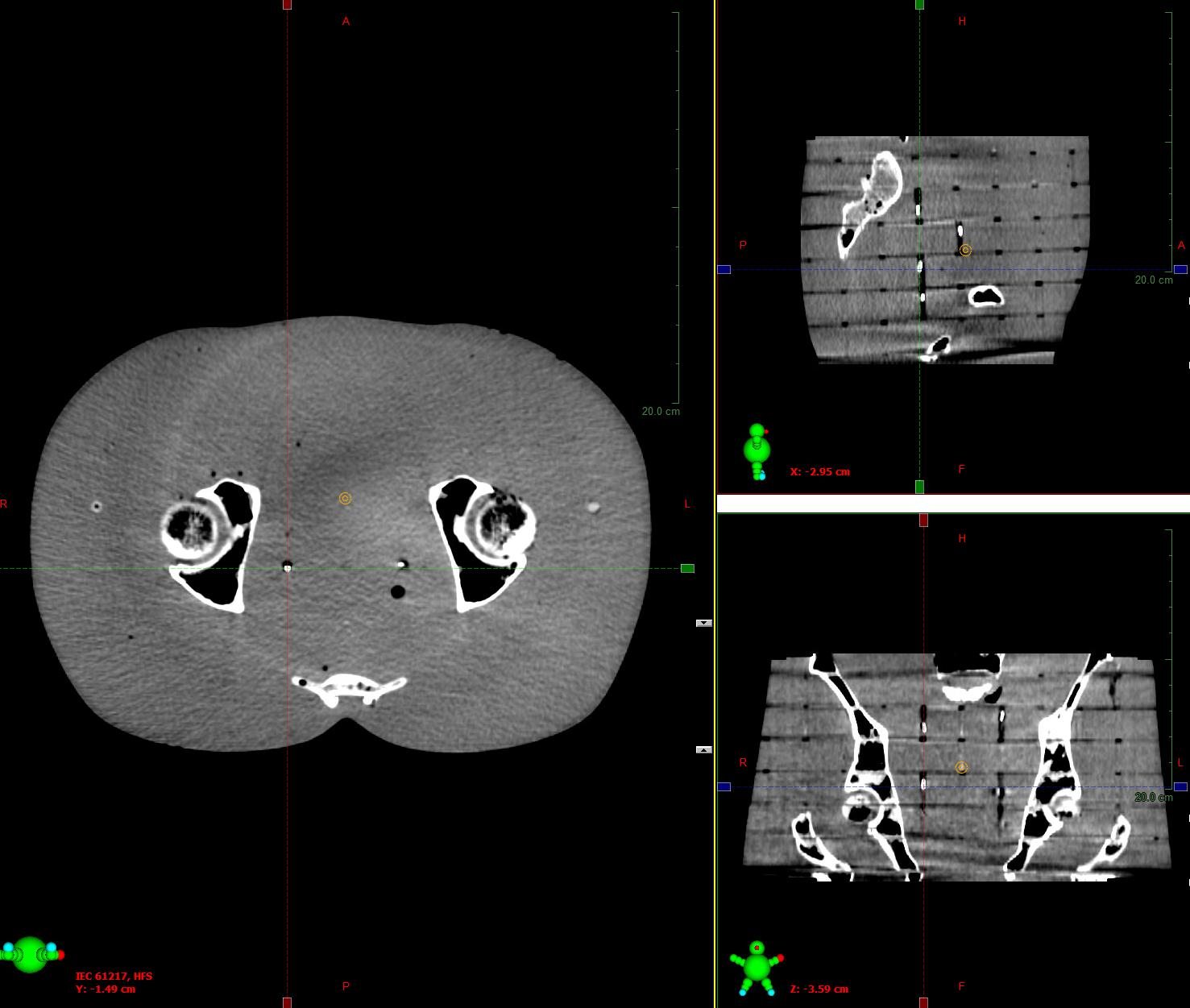

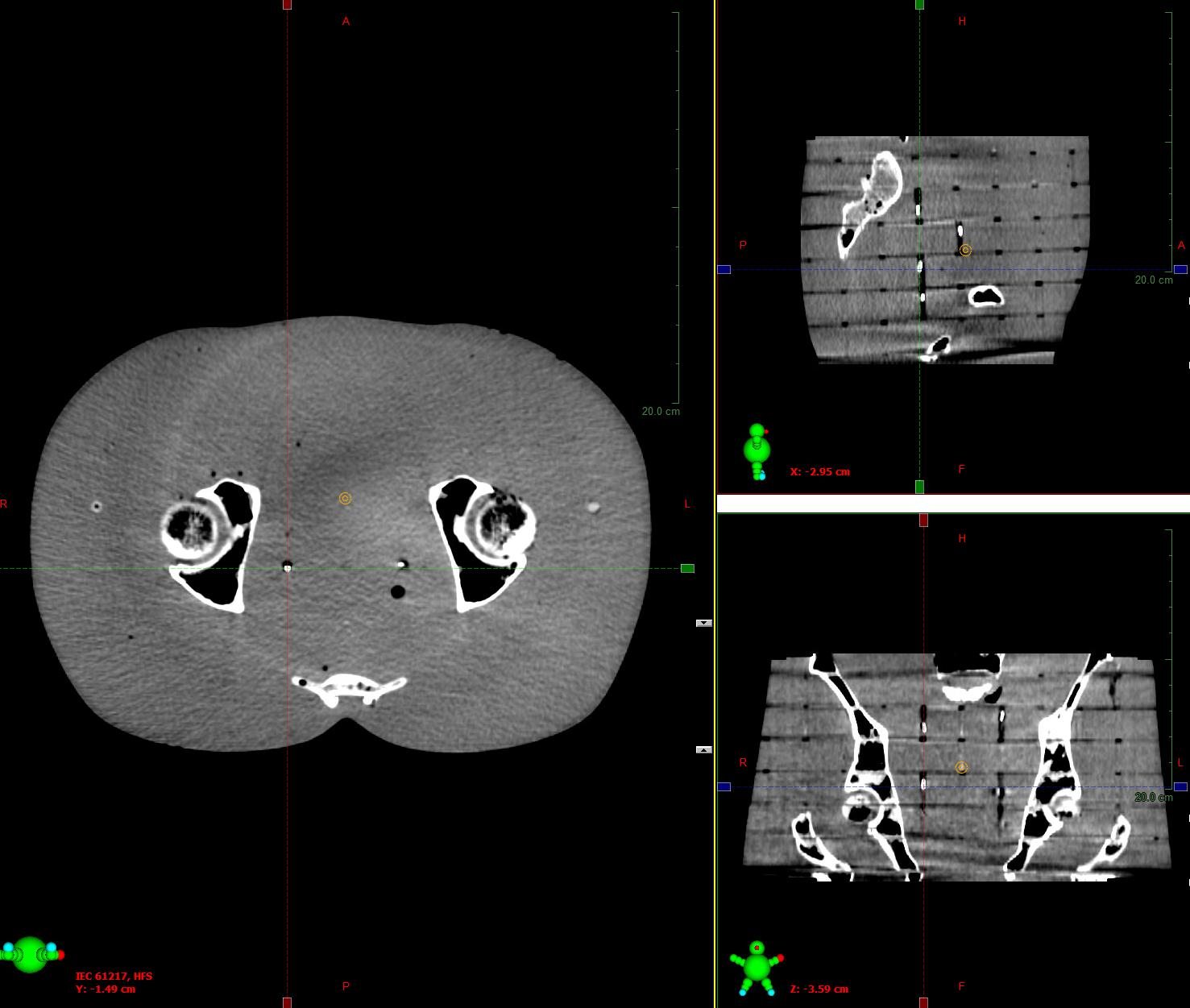


Figure S2.2. Three planar view of CBCT that was acquired using the standard Pelvis protocol (125kVp/60mA/17.9s). All fiducial markers were readily identifiable on the corresponding image slices.

**Supplement S3.** Proton plan used for perturbation measurement.

| (a)  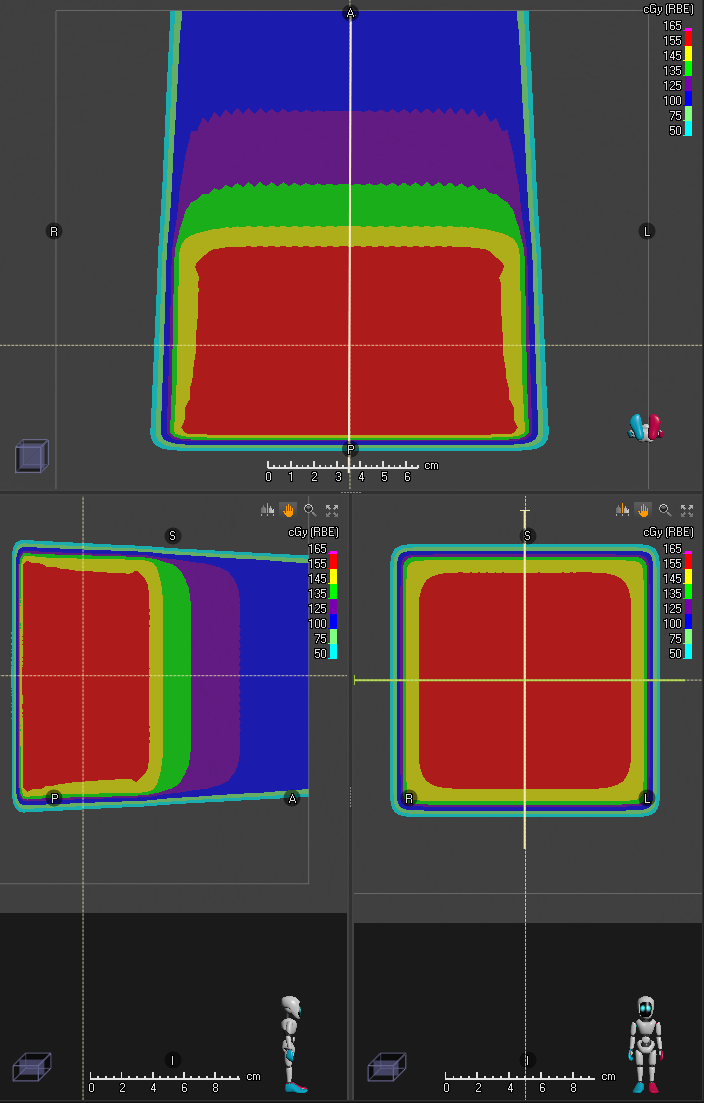 | (b)  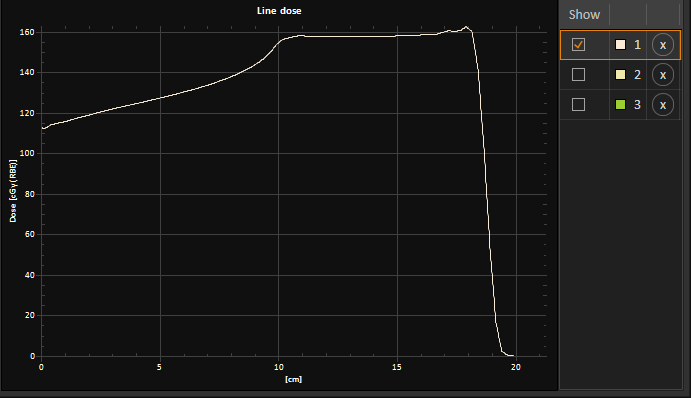 |
| --- | --- |
|  | (c)  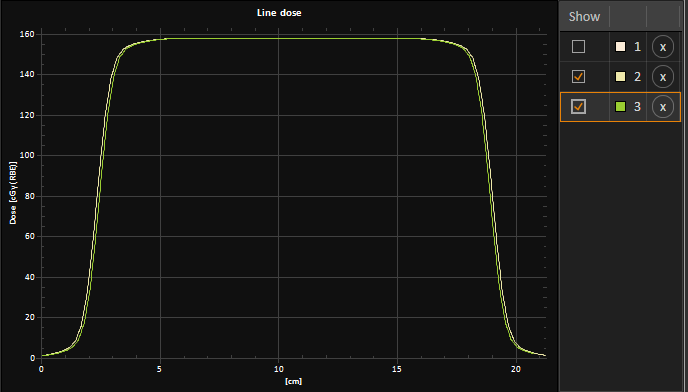 |

Figure S3. (a) dose distribution of the R18M8 plan. (b) depth dose curve along the central axis. (c) dose profiles across the central plane of the SOBP.

The plan was initially optimized using inverse planning in RayStation to achieve dose uniformity across the SOBP (R18M8). Then, for each energy layer, the MUs of all spots were adjusted to be the same and match the average MU per spot. Each energy layer consists of 961 spots with an equal spot spacing of 0.5 cm to cover a field size of 15x15cm^2^. The resulting SOBP flatness is approximately 5%, and high dose uniformity is maintained in the beam profiles. The film measurement region is centered near the central axis, with off-axis distances within 5 cm, where a uniform planar dose distribution is achieved at each depth.
